# Experiencing art creation as a therapeutic intervention to relieve anxiety - a case study of a university’s ceramic art course

**DOI:** 10.3389/fpsyt.2024.1334240

**Published:** 2024-03-04

**Authors:** XiZhi Zhang, Kuohsun Wen, Huan Ding, XiaoHui Zhou

**Affiliations:** ^1^ Faculty of Humanities and Arts, Macau University of Science and Technology, Macao, Macao SAR, China; ^2^ School of Design, Fujian University of Technology, Fuzhou, China; ^3^ School of Design and Innovation, Xiamen University Tan Kah Kee College, Zhangzhou, China

**Keywords:** art therapy intervention, art creation, anxiety, higher education, art and design curriculum

## Abstract

**Background:**

University students are anxiety prone. Due to their changing their social roles, the proportion of university students with anxiety is relatively high. In this study, using the simple random sampling, we surveyed 53 university students, including sophomores, juniors, and seniors.

**Aims:**

This paper examines the relationship between art creation and anxiety.

**Methods:**

This study uses the Self-Assessment Anxiety Scale (SAS). The test form measures the presence and extent of their anxiety problems through a series of questions. We tested the effects of an art creation process on SAS scores and suggest best practices for course settings and teaching methods for art-related subjects.

**Results:**

Art therapy intervention reduced anxiety. The most effective technique was found to be slapping the clay board during the creation process. Other actions relieved anxiety as well. Results suggest that the art creation process is an application of art therapy effective in relieving anxiety in university students.

**Conclusion:**

Key actions in the process of creating art are closely related to the treatment approaches used in art therapy interventions. This has the potential to not only improve mental health, but also to promote the health and well-being of students. Implications for future research: Rapid societal changes increasing competition for employment creates work and life pressures. University students face challenges with learning, peer competition, and employment, often resulting in anxiety. A diversified curriculum can alleviate anxiety through proper curricular planning and design. Based on this, the university’s arts courses should be able to study how to improve and optimize the existing teaching and learning outcomes and can be integrated with the university’s general education curriculum planning. Through appropriate teaching content and learning methods, the courses of university general education can play a role in reducing students’ anxiety and promote physical and mental health, thus contributing to sustainable development of the society.

## 1 Introduction

The rapid pace of development and the complexities of modern life have led to a marked increase in anxiety. According to the World Health Organization (WHO), as if 2019, approximately 301million people globally were affected by anxiety disorders. Recent data indicates that a significant proportion of the global population, irrespective of geographical boundaries, struggles with anxiety disorders. According to data released by China’s State Council Healthcare Commission in 2019, nearly five percent of the Chinese population suffer from anxiety disorders. The prevalence of depression and anxiety disorders is close to seven percent.

University students are particularly prone to anxiety. In the case of current Chinese university students, they must face the pressure of employment after graduation, changing family dynamics and social expectations. And the number of new students going to university has increased over the years. In 2021, 9.09 million college graduates will graduate, an increase of 350,000 over the previous year. In 2022, the number of university graduates is expected to increase by 1.67 million, setting a new record high (1). According to the official announcement of China’s Ministry of Education 2023 college graduates have reached 11.58 million. These increases the competing for employment which may be a psychological stressor for students in China (2). Students’ mental health is critical for sustainable development. Several studies indicate that increases in employment pressure increases depression and anxiety they encounter (3). Tension over employment impacts students’ anxiety (4). During COVID, anxiety levels of university was higher than usual with over 28 percent showing serious anxiety symptom (5). Art therapy is an effective way to prevent or alleviate anxiety (6). At the university level, if learning processes can reduce anxiety, it will help to improve student physical and mental health.

Anxiety is a complex, unpleasant psychological state involving nervousness, unease, worry, and fear of upcoming dangers or threats. It is a negative emotional experience generated when one is unable to cope with perceived threats (7). Barlow (8) further defined anxiety as a cognitive-emotional structure referring to the cognition of hopelessness and uncontrollable feelings of potential danger or threat, as well as a series of worries arising from being alert to potential threat signals. Although moderate anxiety can stimulate learning potential, a chronic anxiety state can cause serious physical and mental damage. Freud believed that anxiety is an experience of tension or fear, an unconscious instinctive impulse. Those experiencing anxiety cannot clearly state the specific objects that make them nervous and worried (9). External interventions such as art therapy can mitigate feeling of anxiety.

Art therapy has value for alleviating anxiety in university students. Art therapy originated in Europe and America after WWII and has gradually grown into an interdisciplinary professional based on theories of art, psychology, philosophy, sociology, and other fields. The treatment emphasizes that visual symbols are the most natural form of communication in the human experience. Therapy involves the client and professional art therapist working to complete various art creation tasks, such as painting and music, with the goals of establishing a professional relationship and carrying out multi-angle interactive actions around artworks (10). Chen (11) used painting therapy with patients with schizophrenia and found that patients’ depression and action levels significantly improved following treatment. Qiu et al. (12) reviewed studies on the role of painting therapy in psychological rehabilitation and found that therapy did lead to improved mental states. Current applications of painting therapy target schizophrenia, depression, autism, and other physical and mental illnesses.

Wang (13) examined depression in university students and found that after eight weeks, painting therapy improved their depression, self-understanding, understanding of others, communication skills, and sense of responsibility. Wang (14) implemented drawing therapy in adolescents with depression and found that following the intervention negative emotions, positive emotions, and self-esteem improved. For art related courses, art therapy both add value and contributes to sustainable development in higher education settings.

In the realm of therapeutic pedagogy, art therapy stands as a testament to the value of creative expression and psychological introspection. The use of painting, drawing, and sculpting and clay modelling as these creative practices focus on the process of creating and experiencing aimed to facilitate the expression of memories, feelings, emotions, to improve self-reflection, and to develop and practice new coping skills (15). Creating art usually requires an individual to be fully engaged in the moment and enter a state of positive thinking. When fully engaged in the creation process, the brain focuses on the task at hand, diverting attention away from anxious thoughts and worries. Engaging in creative acts activates the release of endorphins, dopamine, and serotonin. These neurochemicals promote feelings of pleasure, relaxation, and well-being. By treating anxiety with art therapy, individuals can experience a catharsis of repressed emotions, resulting in a sense of relief, and improved emotional well-being.

Cantu and Fleuriet (16) found that professionally taught arts programs promote a sense of well-being and contribute to brain health by promoting enhanced the ability to concentrate. Proficiency in hands-on artistic pursuits works to ameliorate anxiety symptoms, suggesting that artistic expression promotes emotional well-being. During art therapy, participants engage in a process that focuses concentration, catalyses divergent thinking, and unfolds practical craftsmanship, relieving internal stressors. Thus, when integrating art therapy into university curriculum, a strategic emphasis assumes significance. Within the pedagogy, the cultivation of active interplay between educators and students, emboldens an organic synergy that invigorates insightful contemplation.

As academia continues to evolve, the integration of therapeutic modalities within curricular frameworks emerges as a strategy for fostering holistic student development. In the professional education and general education of the university, adding subjects with art creation tasks is of positive significance to help students regulate and reduce anxiety in different learning stages.

## 2 Methods

A university-level ceramic art course serves as a case study to explore the therapeutic benefits of art creation in mitigating anxiety. Since the object of this study is university students, the main test question is whether the creation of art course can alleviate their anxiety, so the sample of the study adopts the simple random sampling method and the course consists of a class of 53 students in the second, third and fourth years who are tested during the taught study period. The learning objectives aims to equip students with foundational skills in ceramic art creation, including hand-building, wheel-throwing, and sculpting techniques. Additional objectives focus on familiarizing students with the properties and selection of ceramic materials, ensuring adept utilization of tools and equipment. Ultimately, through the creation process, the course seeks to cultivate creative thinking and artistic sensibilities, enabling students to create tangible ceramic art.

The pedagogical blueprint for the ceramic art course includes theoretical instruction, hands-on practice, creative exploration, and reflective exhibition. Through hands-on activities, instructors provide introductions to material, demonstrations, interactive creation sessions, and opportunities to share insights sharing. During the interactive creation phase, students engage in six pivotal practical steps including kneading the clay, slapping the clay slab, repeated rubbing the clay rolls, merging clay with clay, painting greenware, and decorative carving. These steps in the creation process could immerse students in the art, emphasizing the therapeutic potential of ceramic art as a way to relieve anxiety.

### 2.1 Study design

This study used the Self-Rating Anxiety Scale (SAS) to measure anxiety. The scale was originally developed by Zung (17) in 1971 to evaluate the subjective feelings of individuals with anxiety disorders. It is a widely used tool for adults and adolescents. Tao and Gao found that SAS had good reliability and validity based on a sample of patients various neurological conditions. They that the SAS is a better modified version to the Anxiety Clinical Symptom’s Self-Rating Scale, and it is widely used in clinical and mental health fields (18).

Art therapy focuses on experiencing key actions in art creation process. The course examined for this study addressed ceramic art creation. The course focuses on creation of art with clay materials. Clay is an effective medium in art therapy because it promotes an experience that is particularly suited to immersive engagement (19). From an art therapy perspective, the therapeutic process creates a visual or tactile image assisting patients with contemplating actions, the creating the image, and searching for meaning in the process (20). These processes create opportunities for mental and physical movement for clients to boost awareness of gaps in thinking and emotions. It allows clients to see, clarify, and interpret their emotions, beliefs, and life practices from many perspectives. Art therapy emphasizes connections between art and people’s internal images, which is different from normal counselling and psychology. Consequently, the ceramic art creation process produces a form of therapy that is more easily accepted than direct expression of words.

**Figure 1:**
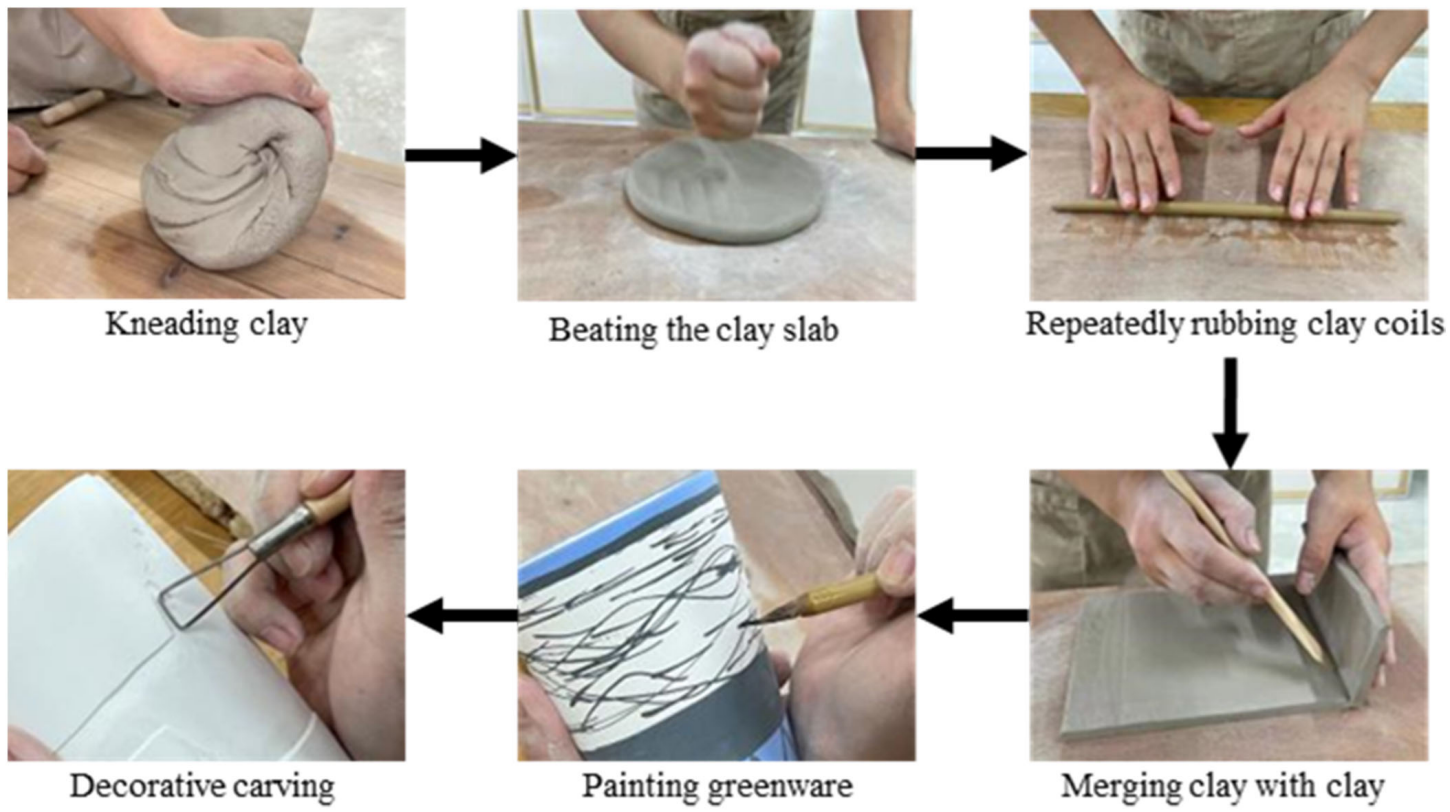
Sequential steps of six key actions of ceramic art creation.

The aim of this study is to examine how a creation process involving hand-kneading ceramics influences anxiety. Participants cut, pile, knead, rub, and squeeze clay with their hands. The hand-kneading technique emphasizes the natural characteristics of clay and the expressiveness of shape. The ceramic art construction mainly includes the coil, the clay slab, the decorative approach containing color painting, and engraving (21). Through literature reviews and observations, we identified six actions central to the study. They are kneading the clay, slapping the clay slab, repeated rubbing the clay rolls, merging clay with clay, painting greenware, and decorative carving (**Figure 1**). Students involved in the art creation process experience the six main actions in order.

### 2.2 Intervention description

Before implementing the study, the nature of the study, the process and goals of ceramic art creation were explained to ensure all participants are fully informed and agreed to participate and complete the anxiety self-rating assessment scale. Afterwards, the completed influential factor test scale was examined.

#### 2.2.1 Stage 1

First, participants received a piece of clay and a suitable amount of clay was placed in their hand. Participants kneaded the clay in circles by hand, shaping round and uniform balls. Participants next placed the balls on a wooden board and crushed into flat shape with consistent thickness and then flattened the clay slab with a rolling pin to achieve desired thickness. They then used a ceramic art tool to trim the clay slab to the desired shape. Finally, the clay slab was merged with the other, producing a three-dimensional piece of ceramic artwork.

#### 2.2.2 Stage 2

Mirroring the procedures in Stage 1, the clay was kneaded into balls and put on the table. Students then smash these balls into flat shape and cut them it into the expected bottom shape. Next participants, rubbed up and down on the clay with their hands making the clay into long coils. Then students bent the clay into coils.

#### 2.2.3 Stage 3

Participants started decorating the ceramic artwork through painting or carving. Before painting, they sketched the shape frame on the greenware used high-temperature paint to draw outlines. Participants then colored and engraved the shapes.

### 2.3 Participants

53 students from Macau University of Science and Technology participated. They were third- and fourth-year undergraduate students aged between 21 – 23.

### 2.4 Measurements and Materials

#### 2.4.1 The usage of anxiety self-assessment scale

As shown in **Table 1**, participants completed SAS before and after the activity (22). The scale includes 20 items measured on a four-point scale.

**Table 1:** Self-Assessment Anxiety Scale.

No.	Self-Assessment Anxiety Scale	1	2	3	4
1	I feel more nervous and anxious than usual (anxiety)	○	○	○	○
2	I am afraid for no reason (fear)	○	○	○	○
3	I am easily upset or frightened (panic)	○	○	○	○
4	I think I might be going crazy (crazy feeling)	○	○	○	○
5	I think everything is fine and nothing unfortunate will happen (unfortunate premonition)	○	○	○	○
6	My hands and feet are shaking (hands and feet are shaking)	○	○	○	○
7	I am distressed by headache, neck pain, and back pain (body pain)	○	○	○	○
8	I feel weak and tired easily (asthenia)	○	○	○	○
9	I feel calm and can easily sit quietly (inability to sit still)	○	○	○	○
10	I feel like my heart is slapping fast (palpitations)	○	○	○	○
11	I am troubled by bouts of dizziness (faintness)	○	○	○	○
12	I have fainted or feel like I am going to pass out (fainting sensation)	○	○	○	○
13	It is easy for me to breathe in and out (difficulty breathing)	○	○	○	○
14	Numbness and tingling in my hands and feet (tingling in my hands and feet)	○	○	○	○
15	I suffer from stomach pain and indigestion (stomach pain or indigestion)	○	○	○	○
16	I have to urinate a lot (frequent urination)	○	○	○	○
17	My hands are often dry and warm (sweaty)	○	○	○	○
18	My face is red and hot (facial hot flashes)	○	○	○	○
19	I fall asleep easily and have a good night’s sleep (sleep disorder)	○	○	○	○
20	I have nightmares (nightmare)	○	○	○	○

### 2.5 Procedures and ethics

#### 2.5.1 Ceramic art therapy techniques applied in the intervention process

The ceramic art therapy intervention invited participants to create art with clay, experience the material tactilely, and explore connection between their emotions and the clay. Before starting, the instructor explained the steps. Participants went through each step, while projecting their feelings and emotions projected onto artistic product.

#### 2.5.2 Participant ratings

Following completion of 45 hours, the measuring scale indicated on a scale from 1 (*weakest*) to 5 (*strongest*) how helpful each of the six activities was in reducing anxiety.

## 3 Results

The ceramic creation process improved university students’ physical and mental health. This study applied EpiData for data entry, and SPSS 24.0 statistical software for data analysis. The study analyzed differences from pre-test to post-test to assess the effects of the intervention.

### 3.1 Test analysis of self-assessment anxiety scale in each dimension

As shown in **Table 2**, comparisons of SAS scores before and after the intervention found that anxiety decreased from before the intervention *M* = 52.9, *SD* = 12.1 to after the intervention *M* = 47.9, *SD* = 4, t (df), = 2.67, p = .01. The result support that ceramic art creation relieves anxiety.

**Table 2:** Test data of SAS in each dimension.

Variable	Group	M	SD	T-test	Sig.
Anxiety value	Pre-test	52.9481	12.09710	2.672	0.010
	Post-test	47.9009	9.35020		

#### 3.1.1 Comparative analysis of pre-and post-test data of ceramic art creation using SAS

As shown in **Table 3**, we examined whether categorization of student anxiety changed from pre to post-test. At pre-tests, 39.6% of the participants did not have anxiety, 37.7% had mild anxiety, 13.2% had moderate anxiety, and 9.4% had severe anxiety. After the intervention, 64.2% did not evidence anxiety, 20.8% were mildly anxious, 15.1% were moderately anxious, and 0.0% were severely anxious. Anxiety decreased after the intervention, χ^2^ (3) = 10.75, p = .013.

**Table 3:** Anxiety self-assessment rating before and after ceramic art creation.

Group	No anxiety	Mild anxiety	Moderate anxiety	Severe anxiety
Pre-ceramic art creation	21 (39.6%)	20 (37.7%)	7 (13.2%)	5 (9.4%)
Post-ceramic art creation	34 (64.2%)	11 (20.8%)	8 (15.1%)	0 (0%)
	10.752			
P	.013			

#### 3.1.2 Analysis of the influential factor test data of ceramic art creation process

**Table 4** represents ratings of how much each activity reduced anxiety. The influential levels of “kneading clay” were as follows: 1 (17.65%), 2 (7.84%), 3 (21.57%), 4 (25.49%), and 5 (27.45%), “slapping the clay slab”: 1 (13.73%), 2 (7.84%), 3 (19.61%), 4 (27.45%), and 5 (31.37%), “repeated rubbing the clay rolls”: 1 (21.57%), 2 (15.69%), 3 (27.45%), 4 (17.65%), and 5 (17.65%), “merging clay with clay”: 1 (19.61%), 2 (19.61%), 3 (21.57%), 4 (9.8%), and 5 (29.41%), “painting the greenware”: 1 (13.73%), 2 (7.84%), 3 (21.57%), 4 (29.41%), and 5 (27.45%), and “carving the greenware” were: 1 (17.65%), 2 (1.96%), 3 (29.41%), 4 (29.41%), and 5 (21.57%). Slapping the clay slab was rated as the most effective for mitigating anxiety. For verifying their validity, following *t* test data analysis was executed.

**Table 4:** Test data analysis of the influential factors of experiencing ceramic art creation.

Actions	1	2	3	4	5
Kneading the clay	17.65%	7.84%	21.57%	25.49%	27.45%
Slapping the clay slab	13.73%	7.84%	19.61%	27.45%	31.37%
Repeated rubbing the clay rolls	21.57%	15.69%	27.45%	17.65%	17.65%
Combining clay with mud	19.61%	19.61%	21.57%	9.8%	29.41%
Painting greenware	13.73%	7.84%	21.57%	29.41%	27.45%
Carving greenware	17.65%	1.96%	29.41%	29.41%	21.57%

#### 3.1.3 Differential analysis of the influence on experiencing ceramic art creation process on emotions

Average scores for each stage were are show in **Table 5** with paired t-tests comparing each mean. The results indicating that slapping the clay was rated higher at reducing anxiety than kneading clay, rubbing the clay rolls, merging the clay, painting, and carving. Participant also rated rubbing clay as less effective at reducing anxiety than kneading the clay and painting greenware.

**Table 5:** Differential analysis of the influence of experiencing ceramic art creation actions on emotions.

	Mean	Std Dev	1	2	3	4	5	6
1	3.38	1.404						
2	3.81	1.345	.434					
3	2.96	1.372	−.415*	−.849**				
4	3.13	1.507	−.245	−.679*	.170			
5	3.49	1.325	.113	−.321	.528*	.358		
6	3.36	1.317	−.019	−.453	.396	.226	−.132	

* p<.05; ** p<.01.

1. kneading clay; 2. slapping the clay slab; 3. repeated rubbing the clay rolls; 4. merging clay with clay; 5. painting greenware; 6. decorative carving of greenware.

## 4 Discussion

University student mental health problems create a multitude of issues. Intervention strategies can effectively enhance resilience and life satisfaction (23). Anxiety and stress are common issues for university students (24). Ceramic art therapy has several unique characteristics, such as materials and craftsmanship. American occupational therapist Arthur Robbins noted that “[c]lay is a special material that gives participants a sense of control and busyness, and in today’s context, it conveys artistic charm.” In ceramic art creation, it is possible to shape and design forms through countless repetitions and applications of external force, pursue the randomness and contingency of textures, and follow processes for hand-made craftsmanship. These approaches improve aesthetic awareness, a central tenant of ceramic art therapy (25).

When experiencing negative emotions, physical actions can positively impact mental health (26). In ceramic art creation, actions like kneading, squeezing, rubbing, pressing, painting, and carving on the clay require specific physical actions that relieve anxiety. The renowned art educator John Dewey was the first to discuss the how making art affects mind and body. In *Art as Experience*, Saint Francis Borgia argued that laborers work only with their hands, but craftspeople use both their hands and brains. In this study, participants worked to create ceramic artwork using their hands, brains, and minds. The process of ceramic art creation involves an information processing discourse based on emotional input. By making handicrafts, participants not only have to handle the material but also obtain sense of belonging. This kind of belonging is what the psychologist Maslow proposed as fulfilling a higher-level need (27).

Art created by handicraft is far more than just production of materials. It is a spiritual pursuit. Ceramic art creation involves delicate and complicated hand crafting. Generally, there are more than 20 production processes and tens of thousands of actions for creating ceramic artwork. The process is completed entirely by hand. The sophistication and complexity of ceramic art is readily apparent. Participants feel a sense of achievement and belonging following completion. During the process, participants are not limited by traditional systems and rules, nor is artwork limited by patterns and symmetry. Participants are free to think boldly and creatively. Using abstract, exaggerated, deformed, and bizarre ceramic art modelling techniques can break out of routine patters of thinking. These processes reduce anxiety. Among the specific tasks, slapping of the clay slab appears to be best way to relieve anxiety.

Psychological interventions are effective treatments for psychological problems (28). Receiving the right psychological counselling improves quality of life (29). Ceramic art therapy applies artistic creative spiritual actions to enhance psychological comfort. Ceramic art involves imagery, manual participation, and freedom in the art creation process.

### 4.1 Incorporating the practice of art creation into the curriculum of university general education

General education (also known as liberal arts education), as defined by Packard is “[a] general education, a classical, literary, scientific, and comprehensive education that prepares students for the study of any profession and provides students with the teaching of all branches of knowledge. This will enable students to have an overall and comprehensive understanding of the overall stages of knowledge before learning specific expertise” (30). General education goals focus on cultivating humanistic qualities, artistic feelings, life care, wisdom, analytical and critical thinking ability, communication skills, lifelong learning, sustainable growth motivation, knowledge, and practice (31). Liberal arts education centers on interdisciplinary knowledge and broad, non-professional, non-utilitarian education as talent development goals (32).

General education plays an important role in the university education system. General education is rooted in historical development and aims to provide students with a comprehensive knowledge base that includes a wide range of subjects. This approach dates to ancient civilizations with the aims to develop critical thinking, communication skills, and a holistic perspective. General education prepares individuals for a rapidly changing world by fostering adaptability and intellectual diversity. By integrating art and design disciplines with the overall goals of general education, educators can tailor programs that not only teach technical skills, but also foster emotional intelligence and provide avenues to relieve anxiety.

The art creation processes discussed in this article usually took place in art-related courses. Art education occupies an important role in general education. Establishing art general education courses is conducive to improving knowledge, aesthetic and humanistic qualities, promoting spirituality, and cultivating and purifying the soul. Art making fosters creative thinking and skills, but also serves as a way to integrate art therapy into the curriculum in a way that can alleviate anxiety. Incorporating thoughtful and structured creative processes into the curriculum creates opportunities to address and alleviate student anxiety. This process requires integrating therapeutic principles into art creation so that students not only gain knowledge and technical skills, but also engage in cathartic outlets for relieving stress and anxiety. This requires careful consideration of course content, implementation strategies, and a home environment conducive to creative work. By strategically integrating art therapy concepts into teaching methods, teachers can help students confront and manage anxiety tendencies.

Integrating artistic creation into the curriculum not only enriches academic experiences, but also helps protect students from the onset and escalation of anxiety states. When general education and art education work synergistically, it can be a powerful tool to strengthen student mental health and promote development. The integration of art and design subjects into academic curricula is a multifaceted endeavor that extends beyond skill acquisition. By incorporating principles of art therapy into the pedagogical approach, students engage in a transformative creative process that serves as a buffer against anxiety. Aligning art and design education with university-level general education goals capitalizes on the historical roots of general education and augments its contemporary relevance. This symbiotic relationship fosters an environment where students not only gain knowledge but also develop tools for managing anxiety.

## 5 Limitations of the study

This study investigated a ceramic art creation intervention with the goal of relieving student anxiety. One limitation is that all participants were students majoring in design. The favorable result of this study suggests opportunities to participant in courses integrating art therapy should extended to different majors. Research on the effects of university curriculum on developing student physical and mental health is insufficient and additional research is needed.

## 6 Implications for further research

There are numerous opportunities for future research focused on issues such as different types of art therapy interventions, investigating mental states of students from different majors, different effects of action and behavior in the art creation process, and curriculum design and implementation. There remains room for exploring methods for using art therapy for mental healing. The scope of application, timing, and usage are promising future developments. Higher education institutions should actively consider how to improve and optimize existing learning methods to integrate activities and content that reduce student anxiety.

## 7 Conclusion

The mental health of Chinese university students is an ongoing concern. Specifically, student anxiety needs to be addressed to ensure the sustainable educational development. This study hypothesized that creating art through ceramics might have a positive effect on anxiety before introducing the methodology. This study examined intersections between art and design and the field of art therapy and explored how to integrate creative processes into therapeutic interventions. This study used a 45-hour art intervention in a three-week design course to provide students insights into the stages of ceramic art creation. Our findings clearly showed that engaging students in basic actions at each creative stage significantly improved anxiety. This confirms our hypothesis that creating ceramic art, with its inherent therapeutic effects, produced a tangible enhancement in mental health.

The art creation process is an effective for integration of art therapy into university curricula. This tailored approach has the dual advantage of fostering artistic creativity and reducing anxiety. This connection is especially important at the university level where students encounter stressful social contexts. Ultimately, the relationship between general education and art creation not only enhances psychological and emotional balance, but also has the potential to have broader effects.

Universities should focus on shifting the prioritizing the experimental aspects of art creation as an important teaching approach in art-related courses rather than focusing too much on the artistic product. Adopting this teaching stance provides the benefits of both, increasing the output of artistic expression, and enhancing mental health. While art therapy is increasingly recognized as a means to reduce anxiety, there remain gaps in the theoretical foundations and practical approaches used to study the effect of art therapy on anxiety.

In conclusion, integrating art and design disciplines into an academic curriculum is a multifaceted endeavor that goes beyond skill acquisition. By incorporating principles of art therapy, students engage in a creative process that buffers against anxiety. Integrating art and design education and general education goals enhances contemporary relevance. This relationship can foster an environment where students gain knowledge and develop a toolkit for managing anxiety and navigating the complexities of modern life. Integration of creative and therapeutic practices is a mechanism for nurturing well-rounded, resilient, and emotionally intelligent individuals.

## Data availability statement

The original contributions presented in the study are included in the article/supplementary material. Further inquiries can be directed to the corresponding author.

## Ethics statement

The studies involving humans were approved by the ethics committee of the Macau University of Science and Technology. The studies were conducted in accordance with the local legislation and institutional requirements. The participants provided their written informed consent to participate in this study.

## Author contributions

XZZ: Conceptualization, Data curation, Formal analysis, Funding acquisition, Investigation, Methodology, Project administration, Resources, Software, Validation, Visualization, Writing – original draft, Writing – review & editing. KW: Conceptualization, Data curation, Methodology, Supervision, Validation, Writing – review & editing. HD: Investigation, Visualization, Writing – review & editing. XHZ: Software, Writing – review & editing.

## References

[1] Ministry of Education of the People’s Republic of China. Xinhua News reports on number of 2021 national college graduates over 9M (2021). Available at: http://www.moe.gov.cn/fbh/live/2021/53931/mtbd/202112/t20211229_591046.html (Accessed Oct 2, 2023).

[2] Che WB Zhang L Huang DM Zhang XD. An investigation on the basic characteristics of College Students’ psychological stress. Appl Psychol. (2003) 9:3–9.

[3] Li R Wen MJ. Analysis on the effectiveness of the course construction of College Students' employment guidance. China Adult Educ. (2017) 19):97–100.

[4] Liu W. The influence of vocational college students’ employment pressure on coping style: The moderating effect of psychological anxiety. Bus Cult. (2020) 35):84–8.

[5] Wang Y. Study on anxiety level of college students and its influencing factors during epidemic prevention and control. J Teach Educ. (2020) 3):76–83.

[6] Ajithakumari MG Hemavathy V. Art therapy reduces anxiety. Anxiety. (90) 2019):46.

[7] Zhang C. Modern Psychology. Shanghai: Shanghai People’s Publishing House (1997).

[8] Barlow DH. Anxiety and Its Disorders: The Nature and Treatment of Anxiety Panic. New York, NY: Guiford Press (1988).

[9] Zhao Y Zhao J. On Freud's anxiety theory. Soc Sci Psychol. (2010) 25:6–24.

[10] Salmon SE Crowley JJ Grogan TM Finley P Pugh RP Barlogie B. Combination chemotherapy, glucocorticoids, and interferon alfa in the treatment of multiple myeloma: A southwest oncology group study. J Clin Oncol. (1994) 12:2405–14. doi: 10.1200/JCO.1994.12.11.2405 7964957

[11] Chen LY. Study on the effect of painting psychotherapy on the rehabilitation of schizophrenic patients. Guangzhou University of Traditional Chinese Medicine, Guangzhou (2015).

[12] Qiu H Liang R Chen L Yi C. Research progress on the role of painting therapy in psychological rehabilitation. Chin J Heal Psychol. (2015) 23:788–92.

[13] Wang CG. Intervention of painting therapy on depression of college students. Nanchang University, Nanchang (2017).

[14] Wang N. Effect of group painting therapy on self-esteem and resilience of adolescent patients with depression. Hebei Medical University, Shijiazhuang (2018).

[15] Malchiodi CA. Handbook of Art Therapy. New York, NY: The Guilford Press (2003).

[16] Cantu AG Fleuriet KJ. “Making the ordinary more extraordinary”: Exploring creativity as a health promotion practice among older adults in a community-based professionally taught arts program. J Holist Nurs. (2017) 36:123–33. doi: 10.1177/0898010117697863 29172944

[17] Zung WK. A rating instrument for anxiety disorders. Psychosomatics. (1971) 12:371–9. doi: 10.1016/S0033-3182(71)71479-0 5172928

[18] Tao M Gao J. Reliability and validity of revised self-rating anxiety scale (sas-cr). Chin J Nerv Ment Dis. (1994) 20:301–3.

[19] Wardi-Zonna K. Finding buddha in the clay studio: Lessons for art therapy. Art Ther. (2020) 37:42–5. doi: 10.1080/07421656.2019.1656459

[20] Yin Y ed. A preliminary study on the mechanism of art therapy. New progress in college mental health education. In: Proceedings of the 10th National Academic Exchange Conference on college mental health education and psychological counseling, China. Chinese Mental Health Association, Harbin. Beijing.

[21] Taylor L Zhao Y Sun S Wang Y. The Ceramic Art Bible. Beijing: Beijing Photography of Fine Arts (2014).

[22] Wang XD Wang XL Ma H. Handbook of Mental Health Assessment Scales. Zhengzhou: Zhengzhou University Press (2008).

[23] Shi M Wang X Bian Y Wang L. The mediating role of resilience in the relationship between stress and life satisfaction among Chinese medical students: A cross-sectional study. BMC Med Educ. (2015) 15:1–7. doi: 10.1186/s12909-015-0297-2 25890167 PMC4332721

[24] Silva RG Figueiredo-Braga M. Evaluation of the relationships among happiness, stress, anxiety, and depression in pharmacy students. Curr Pharm Teach Learn. (2018) 10:903–10. doi: 10.1016/j.cptl.2018.04.002 30236427

[25] Xie X. On the feasibility of ceramic art as a means of art therapy. Decoration. (2009) 12):106–7.

[26] Wang YT Li Z Yang Y Zhong Y Lee SY Chen S. Effects of wheelchair Tai Chi on physical and mental health among elderly with disability. Res Sport Med. (2016) 24:157–70. doi: 10.1080/15438627.2016.1191487 27248716

[27] Hosesar D. History of Psychology. Beijing: People’s Posts and Telecommunications Publishing House (2011).

[28] Schramm E Berger M. Differenzielle indikation für psychotherapie am beispiel der depression. Nervenarzt. (2011) 82:1414–24. doi: 10.1007/s00115-011-3350-3 22051968

[29] Vyas A Babcock Z Kogut S. Impact of depression treatment on health-related quality of life among adults with cancer and depression: A population-level analysis. J Cancer Surviv. (2017) 11:624–33. doi: 10.1007/s11764-017-0635-y 28799098

[30] Li ML Wang YQ. Discussion on the connotation of the concept of “general education”. Tsinghua J Educ. (1999) 101:96–101.

[31] Shih YH. An examination of the functions of general education in a university. Taiwan J Gen Educ. (2014) 2):159–75.

[32] Xue B. Construction of aesthetic education system in universities from the perspective of general education. IETI Trans Soc Sci Humanit. (2019) 5:149–54. doi: 10.6896/IETITSSH.201911_5.0016

